# Association of Body Compositions and Bone Mineral Density in Chinese Children and Adolescents: Compositional Data Analysis

**DOI:** 10.1155/2021/1904343

**Published:** 2021-11-01

**Authors:** Liu Zhang, Hongjuan Li, Yimin Zhang, Zhenxing Kong, Ting Zhang, Zhaohua Zhang

**Affiliations:** School of Sport Science/Key Laboratory of the Ministry of Education of Exercise and Physical Fitness, Beijing Sport University, Beijing 100084, China

## Abstract

The purpose of this study was to investigate the relationship between body compositions and bone mineral density (BMD) and the effect of composition substitution among Chinese children and adolescents without the influence of multicollinearity. A dual-energy X-ray absorptiometry scan was used to determine the amount of truncal fat (TF), nontruncal fat (NTF), fat-free mass (FFM), and BMD. The compositional data analysis and the compositional proportional substitution analysis were conducted to determine the effect of each part of body compositions on BMD and its substitution effects. Four hundred sixty-six (466) (boys: 51.9%) participants completed this cross-sectional study. For girls, in the overweight group, the relationship between TF and the BMD was positive (*β* = 2.943*e* − 01, *p* = 0.006) while the NTF showed the opposite trend (*β* = −2.358*e* − 01, *p* = 0.009). When 4% NTF or FFM was substituted by TF, the BMD increased by about 0.1 and 0.05 units (*p* < 0.05), respectively. For boys, the association between FFM and BMD was statistically positive (*β* = 4.091*e* − 02, *p* = 0.0001). There was a positive correlation between TF and BMD (*β* = 7.963*e* − 02, *p* = 0.036). But with the increase of BMI, this correlation shifted in the opposite direction. In conclusion, compared to TF and NTF, FFM had a better protective effect on BMD, especially for boys. The risk of NTF accumulation on BMD was greater than that of TF accumulation. Compared with girls, boys were more sensitive to the amount of TF.

## 1. Introduction

Osteoporosis has become a global public health problem, causing a huge economic burden on society and reducing the quality of life. Peak bone mass is an important determinant of osteoporotic fracture risk, and adequate peak bone mass is associated with a reduced risk of osteoporotic fracture later in life [1]. Early childhood and adolescence are periods of rapid growth and bone mineral accumulation. At 18, it can reach 90% of the individual's peak bone mass [2]. According to statistics, for every 10% increase in peak bone mass, the risk of osteoporotic fractures in the future will be reduced by 50% or the age of onset of osteoporosis will be delayed by 13 years [3].

Body composition is an important determinant of bone mineral density (BMD), which is mainly composed of two components: fat-free mass (FFM) and fat mass (FM) [4, 5]. Numerous studies have explored the relationship between FFM and BMD and consistently shown a higher percentage of FFM associated with higher BMD because of the mechanical load and the load effect of weight-bearing exercise [6–8]. However, the relationship between FM and BMD is unclear, especially FM distribution and BMD. Traditionally, obesity is considered osteoprotective because excess adipose tissue has a weight-bearing effect on bones [9, 10]. But after adjusting the body weight, some studies found a negative or null association between obesity and BMD [11, 12]. Body fat distribution may also impact BMD. Cao and Pollock et al. pointed out that central obesity hurt bone density [13, 14], whereas Yi et al. believed hip fat was a protective factor [15]. There are limited studies addressing the relationship between fat distribution and BMD, especially in children and adolescents. These restricted prior observations suggest the need to explore the relationship between body composition components and BMD deeply and comprehensively.

Body fat rate or body mass index (BMI) is often used as a surrogate measure to reflect overweight and obesity; however, it cannot provide accurate information about the distribution of body fat (e.g., Android fat or Gynoid fat), nor can it distinguish whether body weight comes from fat, muscle, or bone. Compositional data comprises mutually exclusive and exhaustive parts that add up to a total of 100% or 1 [16]. For example, body compositions, consisting of truncal fat (TF), nontruncal fat (NTF), and FFM, have the characteristics of compositional data. Compositional data analysis (CoDA) is a statistical technique designed to properly model the association between compositional variables and the outcomes. Because of calculating the original absolute data to obtain the proportional structure, CoDA can further explain the relative information behind the absolute data. However, the constant sum constraint increases the multicollinearity among components [17, 18]. To avoid the influence of multicollinearity and retain all the simplex measurement attributes, Egozcue et al. proposed the isometric log ratio (ILR), which was converted by constructing a standard orthogonal basis to make a compositional data map from the simplex space SD to the Euclidean space RD-1 [19]. Therefore, considering that each part of the body composition of children and adolescents is closely related, it is more reasonable to analyze the components as a whole under the CoDA.

Because of the critical period of bone growth in children and adolescents and the limited studies, understanding the impact of the constitution of body compositions and those distributions on bone health is important when planning strategies to prevent fractures and osteoporosis later in life. Therefore, by using CoDA (ILR transformation),which can avoid multicollinearity, this study is aimed at examining the association between body compositions (%TF, %NTF, and %FFM) and BMD and the substitution effects when reallocating each part to others in Chinese children and adolescents.

## 2. Materials and Methods

### 2.1. Study Population and Design

The present study is a cross-sectional study, and the samples of school children are nonrepresentative. Recruitment of participants was performed on the school level. In the spring of 2010, the project leader contacted 16 schools in Beijing, China, and presented information about the study. Each student from the recruited classes received a letter with detailed information regarding the purposes and methods of the study, and the parents needed to sign the informed consent. Measurements took place between April and July 2010. The inclusion criteria of the research objects are as follows: (1) complete basic information (age and sex) at baseline and (2) complete all test items. Exclusion criteria: (1) participants with a history of diabetes, kidney disease, and other acute or chronic diseases and (2) limb disability. In total, 473 children aged 5–18 years voluntarily participated in this study, and the final effective sample size was 466.

### 2.2. DXA Measurements

A dual-energy X-ray absorptiometry scan (DXA) was used to test all subjects' body compositions (TF, NTF, and FFM) and BMD (whole-body bone mineral density, upper-limb bone mineral density, thigh bone mineral density, trunk bone mineral density, and pelvic bone mineral density). The body mass was calculated as FFM + FM. We used the same DXA machine for all participants, calibrating machine drift daily with the standard synthetic phantom. Each measurement was taken by the well-trained operators.

### 2.3. Anthropometric Measurements

Height was assessed to the nearest 0.1 cm using a portable stadiometer by trained research staff. Body mass index (BMI) was calculated as body mass (in kilograms) divided by the square of height (in meters). According to the BMI classification standard for overweight and obesity of school-age children and adolescents in China [20] and screening standard for malnutrition of school-age children and adolescents for a specific age and gender [21], subjects were divided into four groups: underweight, normal weight, overweight, and obesity.

### 2.4. Statistical Analysis

The first step of CoDA was to calculate the proportion of body compositions (%TF, %NTF, and %FFM). Then, the ratios were expressed as isometric log-ratio coordinates using ILR. Therefore, conventional descriptive statistics can be applied. The geometric mean was used as the central tendency of each component of the body composition, which can better show the center of the composition data point [22]. The ternary plot can visualize three components. The further away from the vertex indicates the smaller the proportion. The variation matrix was used to describe the discrete trend of CoDA. The more the value is connected to zero, the stronger the correlation between the corresponding two components. The compositional multiple regression model was used to analyze the relationship between body compositions and BMD of each part in different BMI groups; the compositional proportional substitution model was used to quantify the reallocation effects of body composition and BMD. Covariates include age and body mass. Analyses were performed in R 3.6.3 using the “compositions” package. *p* values <0.05 were considered statistically significant.

## 3. Results

### 3.1. Descriptive Analysis

Four hundred sixty-six participants (51.93% male) with completed data were included in this study. The mean age was 12.23 (3.13) years. Most (62.66%) of the subjects were categorized as normal weight, and the proportion of overweight and obesity was 25.75%. The geometric mean values of the three components of body composition are shown in Table 1.

### 3.2. Body Composition Variation Matrix

The variation matrix (Table 2) calculated the variance in the log ratio of two parts. The variation matrix value between TF and NTF was the smallest (0.06). The larger values between TF, NTF, and FFM (0.46 and 0.33) showed that there was a small correlation between fat mass and fat-free mass.

### 3.3. Results of Ternary Graphs for Different Groups

**Figure 1:**
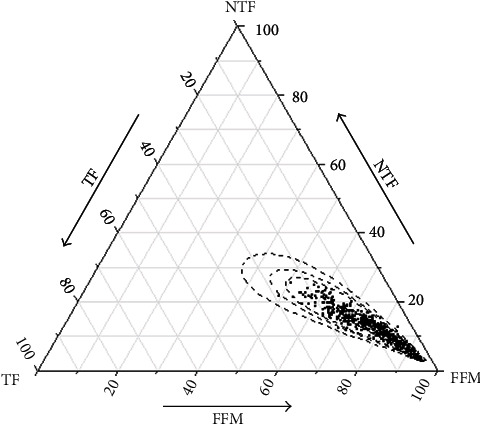
Relative information for body compositions is surrounded by 90%, 95%, and 99% normal probability areas.

**Figure 2:**
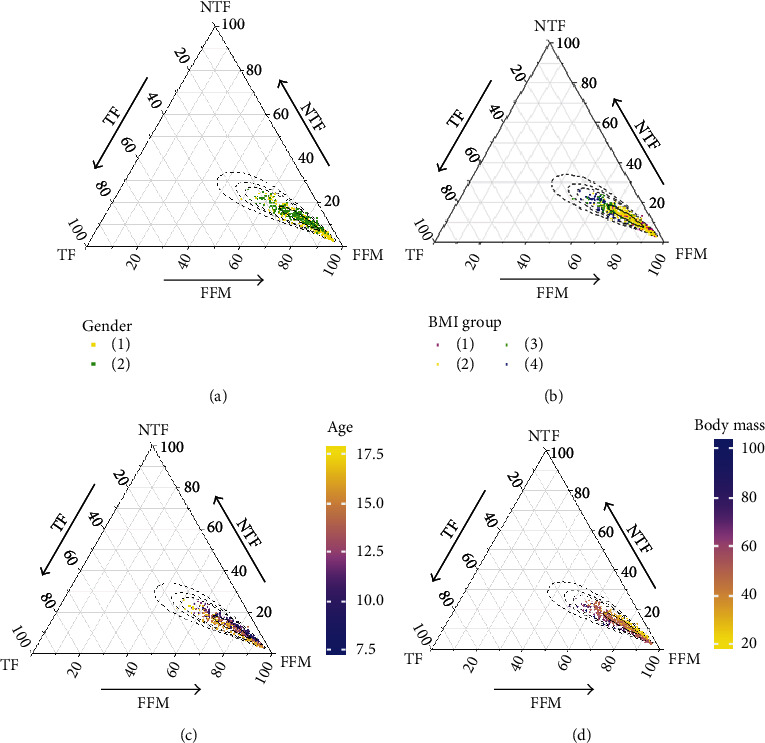
There is relative information of body compositions in different groups, surrounded by 90%, 95%, and 99% normal probability areas. (a) Gender group, 1: male and 2: female; (b) BMI group, 1: thin, 2: normal weight, 3: overweight, and 4: obesity; (c) age distribution; (d) body mass distribution.

In this study, the body composition data was divided into three parts to be perfectly plotted in the ternary diagram. The distribution of data points in Figure 1 suggested that as the proportion of FFM increased (moving towards the peak of the triangle), the proportions of TF and NTF decreased more equally.  Figure 2(a) shows the gender difference in the body compositions of the study subjects. Compared with girls, boys had a lower proportion of fat mass and a higher proportion of FFM. The points of children and adolescents in the underweight group and the normal-weight group were mostly concentrated at the tip of the triangle, composed of a lower proportion of adipose mass and a higher proportion of FFM (Figure 2(b)). With the increase of age and weight (Figures 2(c) and 2(d)), the proportion of TF showed an upward trend, and the proportion of NTF continued to decrease. Therefore, the next main analyses were discussed by gender, with age and body mass used as covariates.

### 3.4. Compositional Multiple Regression Analysis

Tables 3 and 4 present the association between the body compositions and BMD after adjusting for age and body mass.  Table 3 shows the results of girls. For girls in the overweight group, the relationship between TF and the BMD of each part was positive, while the NTF showed the opposite trend (*p* < 0.01). For normal-weight children, NTF showed a significant negative correlation with trunk and pelvic BMD (*p* < 0.01). There was no significant correlation between FFM and whole-body BMD (*p* = 0.171). However, when we considered each part of the BMD, the relationships between FFM and BMD of upper limbs, thighs, trunk, and pelvis were statistically positive (*p* < 0.05).

For boys in Table 4, in addition to the BMD of the thigh, TF showed a strong positive correlation with the bone mineral density of the whole body, upper limbs, trunk, and pelvis (*p* < 0.05), especially in the normal-weight group. The trend of the accumulation of TF in different BMI groups was worthy of our attention. These trends showed a strong regularity in a different part of BMD. Taking whole-body BMD as an example, there was a positive correlation between TF and BMD (*β* = 7.963*e* − 02, *p* = 0.03602). With the increase of BMI, this protective effect gradually weakened until the obesity group began to show a negative correlation trend (*β* = −2.051*e* − 01, *p* = 0.09448). There was a significant negative correlation between NTF and most BMD (*p* < 0.05). And the higher FFM was associated with higher BMD (*p* < 0.05).

### 3.5. Compositional Proportional Substitution Analysis

**Figure 3:**
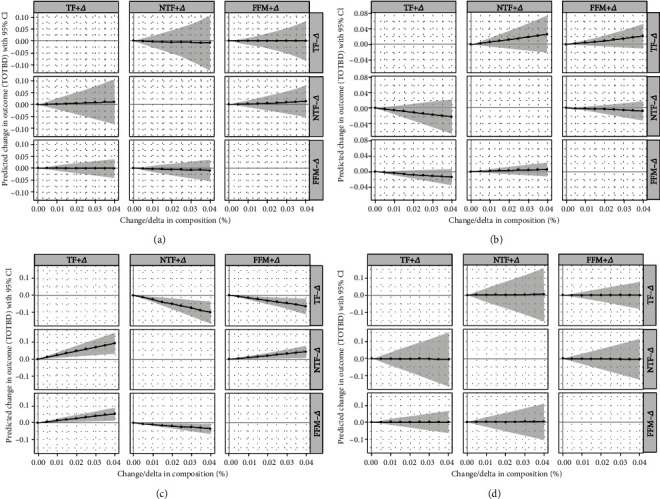
The results of compositional substitution analysis—girls ((a) underweight group; (b) normal-weight group; (c) overweight group; (d) obesity group); BMD: bone mineral density.

**Figure 4:**
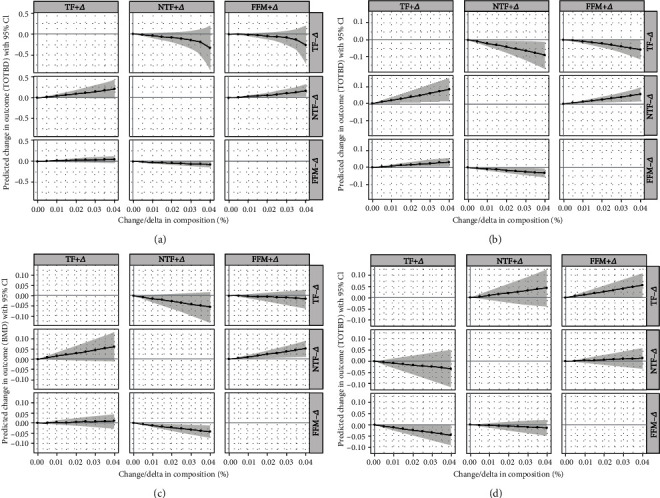
The results of compositional substitution analysis—boys ((a) underweight group; (b) normal-weight group; (c) overweight group; (d) obesity group); BMD: bone mineral density.

Based on the compositional multiple regression model, the geometric mean of each component of body compositions was used as the reference value. When the total amount remained the same, Figures 3 and 4 showed that each component of the body compositions was increased by 1% to 4% in a “one by one” redistribution manner to analyze its effects on BMD. For girls in the overweight group (Figure 3), when TF substituted 4% NTF or FFM, the BMD increased by about 0.1 and 0.05 units (*p* < 0.05), respectively. When FFM increased (from 1% to 4%) and NTF decreased, BMD showed a downward trend, approximately 0.5 units (*p* < 0.05).

The redistribution of body composition in boys was different from girls (Figure 4). The proportion of TF increased from 1% to 4%, corresponding reduction in NTF or FFM by 1%–4%; the BMD showed an upward trend in the underweight, normal-weight, and overweight groups, especially in the normal-weight group, about 0.08, 0.04, units (*p* < 0.05), respectively. As for the obesity group, though there was no statistical significance, the higher proportion of TF related to the lower BMD. When NTF was substituted by 4% FFM, BMD increased about 0.05, 0.07 units in the normal-weight and overweight groups.

## 4. Discussion

This is the first study to examine the association between body composition and BMD using CoDA (ILR transformation), especially in children and adolescents. This method considered the influence of each part of the body compositions on BMD and effectively avoided the problem of multicollinearity. In our study, subjects with a higher percentage of FFM had higher BMD, especially for boys. The association between NTF and most BMD was negative. TF was positively associated with BMD in an overweight group in girls and a normal-weight group in boys. Although there was no statistical significance between TF and BMD in the obese group of boys, the excess TF accumulation related to the lower BMD. In addition, this study excluded the influence of body mass on BMD, which will provide a better explanation for the relationship between body compositions and BMD.

Body compositions are typically divided into different components in different studies. Fat mass is often used as a factor affecting health. In this research, the larger variation matrix value between fat mass and FFM further proved that fat mass was often used as an independent factor to explore the relationship between it and health-related indicators. However, changes in fat mass also affect other components. It can be found from the ternary diagram that as age and weight increased, children and adolescents tended to accumulate more TF and less NTF. In a study of 1007 children aged 6 to 15 in Beijing, Li et al. reported that the area of visceral fat would increase with age [23]. Thus, it is very necessary to discuss body composition by gender during this period. In addition, to avoid the influence between the components, Henriksson et al. and Hetherington et al. made mutual corrections for the quality of fat mass and FFM in their studies [24, 25]. Compared with this method, ILR transformation is better to avoid multicollinearity between multiple components [16]. Therefore, in our study, the CoDA (ILR transformation) included all parts of the body composition in the same model according to gender to better understand the relationship between body compositions and BMD.

The concept that higher FFM is always associated with higher BMD has been widely accepted [26]. The results of our study further demonstrate the positive relationship between FFM and BMD in children and adolescents. Because of the common genetic and hormonal determinants [27, 28], muscle and bone are closely related adjacent tissues [29], and loss of muscle mass and bone mass throughout life has been proven to be coupled and has been assumed to be part of the same functional unit [26]. In addition, mechanical loading increases bone formation, while weight-bearing exercise improves bone mineral stress [30]. In other words, a higher FFM represents more muscle mass, meaning bones are loaded through muscle action (functional strain), leading to higher BMD [12]. This effect is more significant among boys because boys have far more FFM than girls.

Beyond FFM, the pattern of fat distribution had different effects on BMD. In our research, NTF had a statistically negative correlation with BMD. In the exercise process, the participation of the limbs is crucial; a higher proportion of muscles are needed. Compared with the functional strain produced by muscles on bones, the static strain produced by fat accumulation has a weak effect on bone strength. At the same time, both adipocytes and osteoblasts share a common progenitor. These stem cells are regulated by common factors, such as PPAR-*γ*, in the process of differentiation into osteoblasts and adipocytes. Studies have shown that PPAR-*γ* is highly expressed in adipose tissue, making bone tissue more absorptive than bone formation, leading to osteoporosis [13, 31].

TF was positively correlated with BMD in the overweight group in girls and the normal-weight group in boys. Inconsistent with our finding, the majority of previous studies involving children and adolescents [8, 32, 33] or adults [27, 34] have reported a negative relationship between TF and BMD. However, unlike our research with a wide range of body and fat mass, most of these studies were conducted in overweight or obese children. Though no significant negative correlation between TF and BMD was found in our study, the downward trends of TF in boys deserved our attention. Starting from normal weight, as BMI increased, this positive correlation became weaker and weaker, and a negative correlation began to appear in the obesity group. Rokoff et al., in their research, pointed out that the association between central adiposity and BMD only exited in children with the highest levels of abdominal fat [8]. Another study analyzed the body fat and bone mass of 377 adolescents aged 10–19 years and found that BF% negatively affected bone mass in males, and the higher the BF% was, the lower BMD values in overweight adolescents [35]. Therefore, in children with relatively high-fat content, the higher FM may strongly correlate with lower BMD. Perhaps there was a threshold of TF, after which the individual is more prone to adverse consequences caused by metabolism.

Children and adolescents are in a rapid period of growth and development, and each part of their body composition will change significantly. Unlike adults' more stable body composition, a certain amount of TF accumulation may have a better stimulating effect on bone health. In addition, gender differences also require attention when analyzing the relationship between body compositions and bone mineral density. Puberty is an important milestone in the development of body composition. Most studies show that the total FM% of boys increased before the age of 10–12 and then decreased, while girls' increased gradually with age [36, 37]. Due to the complexity of hormone secretion during this period, boys accumulated more muscle mass and less fat mass and were more sensitive to fat accumulation. Because of less fat mass and higher sensitivity, when the gravity effect caused by fat accumulation is not enough to offset the negative effects caused by the hormone secreted by fat cells, especially abdominal adipose tissue, the adverse effects on bone density will gradually appear [13]. The substitution analysis further verified the above results when relocating body compositions one by one in different groups. Also, it can provide theoretical support for the formulation of precise intervention plans.

This study provided a new methodological perspective for exploring the relationship between body compositions and BMD using CoDA (ILR transformation) in children and adolescents. The compositional proportional substitution analysis can clearly show the effect of fat distribution and body compositions on BMD. We used DXA to measure the body compositions, which can provide accurate data of FFM and fat mass and fat distribution. Also, there are some limitations. First, the overweight and obesity groups of boys and girls are relatively small; future studies should expand the sample size for analysis. Second, we only divide body fat into two parts: TF and NTF. Future research can divide body composition into more parts or other ways (e.g., fat mass, protein, water, and minerals) to deeply explore the relationship between it and BMD. Finally, our cross-sectional data cannot confirm cause and effect.

## 5. Conclusions

Our research demonstrates that FFM has a positive effect on BMD in children and adolescents, especially for boys. The risk of NTF accumulation on BMD was greater than that of TF accumulation. Boys are more sensitive to the amount of TF; excessive TF accumulation may be more harmful to the BMD of boys than girls. Our findings highlight the importance of gender-specific and regional fat influences on BMD and provide a rationale for further exploration of the mechanisms underlying this observed relationship.

## Figures and Tables

**Table 1:** The geometric mean values of the three components of body compositions.

	Boys	Girls	Total
Body composition (%)			
Truncal fat	8.44	10.62	9.45
Nontruncal fat	10.64	13.82	12.09
Fat-free mass	80.92	75.56	78.46

**Table 2:** Body composition variation matrix.

	Truncal fat	Nontruncal fat	Fat-free mass
Truncal fat	0.0000000		
Nontruncal fat	0.06036724	0.00000000	
Fat-free mass	0.46329548	0.33766990	0.00000000

**Table 3:** Multiple linear regression analysis of the relationship between body compositions and BMD (girls—compositional multiple regression model) (*n* = 225).

		Adj *R*^2^	*β*TF	Pr(>∣*t*∣)	*β*NTF	Pr(>∣*t*∣)	*β*FFM	Pr(>∣*t*∣)
Whole-body BMD	1	0.8245	-1.060*e*-04	0.99820	-2.921*e*-02	0.69355	2.932*e*-02	0.60071
	2	0.775	-4.895*e*-02	0.205	2.740*e*-02	0.508	2.154*e*-02	0.220
	3	0.8756	2.943*e*-01	0.006405^∗∗^	-2.358*e*-01	0.009298^∗∗^	-5.857*e*-02	0.365043
	4	0.8626	-2.462*e*-03	0.9904	2.030*e*-02	0.9450	-1.784*e*-02	0.9277
	Total	0.8245	3.704*e*-03	0.889	-2.336*e*-02	0.446	1.966*e*-02	0.171

Upper limb BMD	1	0.7345	3.196*e*-02	0.43857	-2.949*e*-02	0.64873	-2.467*e*-03	0.95962
	2	0.8119	-4.537*e*-03	0.86543	-1.998*e*-02	0.48813	2.452*e*-02	0.04554^∗^
	3	0.908	2.399*e*-01	0.000466^∗∗∗^	-1.718*e*-01	0.00210^∗∗^	-6.807*e*-02	0.084868
	4	0.8403	5.104*e*-03	0.97440	-3.061*e*-01	0.19892	3.010*e*-01	0.07048
	Total	0.8368	3.647*e*-02	0.05973	-6.114*e*-02	0.00646^∗∗^	2.467*e*-02	0.0184^∗^

Thigh BMD	1	0.8514	4.273*e*-02	0.5146	-1.276*e*-01	0.2235	8.489*e*-02	0.281
	2	0.8643	-2.234*e*-02	0.6262	-3.463*e*-02	0.4830	5.697*e*-02	0.00697^∗∗^
	3	0.933	3.561*e*-01	0.00128^∗∗^	-3.927*e*-01	9.23*e*-05^∗∗∗^	3.658*e*-02	0.5629
	4	0.8292	-1.051*e*-01	0.6992	1.735*e*-01	0.6571	-6.832*e*-02	0.7929
	Total	0.8649	9.711*e*-02	0.004807^∗∗^	-1.423*e*-01	0.000369^∗∗∗^	4.519*e*-02	0.014680^∗^

Trunk BMD	1	0.8199	1.304*e*-02	0.7656	-8.246*e*-02	0.2396	6.943*e*-02	0.1913
	2	0.8022	2.831*e*-02	0.37543	-7.176*e*-02	0.0379^∗^	4.344*e*-02	4.344*e*-02
	3	0.9226	3.708*e*-01	0.000115^∗∗∗^	-2.765*e*-01	0.000439^∗∗∗^	-9.437*e*-02	0.077764
	4	0.8669	1.569*e*-01	0.4310	-1.874*e*-02	0.9469	-1.382*e*-01	0.4682
	Total	0.8502	4.448*e*-02	0.050306	-8.599*e*-02	0.00114^∗∗^	4.151*e*-02	0.000778^∗∗∗^

Pelvic BMD	1	0.806	4.171*e*-02	0.5563	-1.487*e*-01	0.1911	1.070*e*-01	0.2115
	2	0.8273	4.608*e*-02	0.3054	-9.748*e*-02	0.0448^∗^	5.140*e*-02	0.0127^∗^
	3	0.8994	4.748*e*-01	0.000652^∗∗∗^	-4.030*e*-01	0.000642^∗∗∗^	-7.183*e*-02	0.361787
	4	0.8021	1.463*e*-01	0.6273	-1.815*e*-02	0.9663	-1.282*e*-01	0.6569
	Total	0.8486	1.002*e*-01	0.00267^∗∗^	-1.493*e*-01	0.000117^∗∗∗^	4.909*e*-02	0.006233^∗∗^

Note: 1—underweight group (*n* = 25), 2—normal-weight group (*n* = 160), 3—overweight group (*n* = 25), and 4—obesity group (*n* = 15); *p* < 0.05^∗^; *p* < 0.005^∗∗^; *p* < 0.0005^∗∗∗^.

**Table 4:** Multiple linear regression analysis of the relationship between body compositions and BMD (boys—compositional multiple regression model) (*n* = 241).

		Adj *R*^2^	*β*TF	Pr(>∣*t*∣)	*β*NTF	Pr(>∣*t*∣)	*β*FFM	Pr(>∣*t*∣)
Whole-body BMD	1	0.5917	8.401*e*-02	0.256432	-1.669*e*-01	0.044409^∗^	8.286*e*-02	0.032967^∗^
	2	0.7837	7.963*e*-02	0.03602^∗^	-1.067*e*-01	0.0107^∗^	2.706*e*-02	0.0478^∗^
	3	0.823	7.971*e*-02	0.34717	-2.032*e*-01	0.0216^∗^	1.235*e*-01	0.000205^∗∗∗^
	4	0.8811	-2.051*e*-01	0.09448	-1.182*e*-02	0.91017	2.169*e*-01	0.00141^∗∗^
	Total	0.7836	6.460*e*-02	0.034039^∗^	-1.055*e*-01	0.00144^∗∗^	4.091*e*-02	0.000108^∗∗∗^

Upper-limb BMD	1	0.8226	4.317*e*-02	0.34878	-1.300*e*-01	0.01422^∗^	8.683*e*-02	0.000887^∗∗∗^
	2	0.8377	0.00391^∗∗^	0.01089^∗^	-8.974*e*-02	0.00391^∗∗^	1.774*e*-02	0.07958
	3	0.7893	1.979*e*-02	0.78945	-1.369*e*-01	0.0743	1.171*e*-01	7.45*e*-05^∗∗∗^
	4	0.8623	-1.091*e*-01	0.35380	-7.304*e*-02	0.47754	1.822*e*-01	0.00500^∗∗^
	Total	0.825	6.663*e*-02	0.00559^∗∗^	-1.055*e*-01	5.7*e*-05^∗∗∗^	3.885*e*-02	3.41*e*-06^∗∗∗^

Thigh BMD	1	0.7791	-4.586*e*-03	0.96242	-1.205*e*-01	0.26162	1.251*e*-01	0.01704^∗^
	2	0.8446	5.854*e*-03	0.9121	-8.195*e*-02	0.160	7.609*e*-02	0.000112^∗∗∗^
	3	0.8674	1.285*e*-01	0.262345	-3.109*e*-01	0.009794^∗∗^	1.823*e*-01	6.2*e*-05^∗∗∗^
	4	0.8265	-3.097*e*-01	0.15855	-5.188*e*-02	0.78379	3.616*e*-01	0.00282^∗∗^
	Total	0.8241	3.416*e*-02	0.440418	-1.062*e*-01	0.027139^∗^	7.199*e*-02	3.51*e*-06^∗∗∗^

Trunk BMD	1	0.701	1.207*e*-01	0.042169^∗^	-2.042*e*-01	0.002757^∗∗^	8.352*e*-02	0.007302^∗∗^
	2	0.841	8.352*e*-02	0.00574^∗∗^	-1.290*e*-01	0.000124^∗∗∗^	4.546*e*-02	4.21*e*-05^∗∗∗^
	3	0.8457	1.698*e*-01	0.0250^∗^	-2.922*e*-01	0.000299^∗∗∗^	1.224*e*-01	3.52*e*-05^∗∗∗^
	4	0.9355	-2.310*e*-02	0.781200	-1.551*e*-01	0.04079^∗^	1.782*e*-01	0.00026^∗∗∗^
	Total	0.8289	9.321*e*-02	0.00021^∗∗∗^	-1.529*e*-01	3.24*e*-08^∗∗∗^	5.967*e*-02	2.17*e*-11^∗∗∗^

Pelvic BMD	1	0.7396	1.429*e*-01	0.102481	-2.938*e*-01	0.00378^∗∗^	1.509*e*-01	0.00168^∗∗^
	2	0.8225	1.184*e*-01	0.016440^∗^	-1.763*e*-01	0.00123^∗∗^	5.799*e*-02	0.00123^∗∗^
	3	0.8425	2.123*e*-01	0.053760	-3.917*e*-01	0.000817^∗∗∗^	1.793*e*-01	3.53*e*-05^∗∗∗^
	4	0.9082	-6.424*e*-02	0.643974	-1.961*e*-01	0.115220	2.603*e*-01	0.001036^∗∗^
	Total	0.8068	1.341*e*-01	0.000846^∗∗∗^	-2.022*e*-01	4.05*e*-06^∗∗∗^	6.808*e*-02	1.08*e*-06^∗∗∗^

Note: 1—underweight group (*n* = 29), 2—normal-weight group (*n* = 132), 3—overweight group (*n* = 48), and 4—obesity group (*n* = 32); *p* < 0.05^∗^; *p* < 0.005^∗∗^; *p* < 0.0005^∗∗∗^.

## Data Availability

The results of the research will be available on request from interested persons (query: zhangliubsu@163.com) or from the corresponding author. This limitation is related to the principles of personal data protection. The data will be free of sensitive data, including the date of birth (age will be available at the time of the examination), name, and surname (the coded number of the respondent will be available).
